# Surgical treatment and prognostic analysis for gastrointestinal stromal tumors (GISTs) of the small intestine: before the era of imatinib mesylate

**DOI:** 10.1186/1471-230X-6-29

**Published:** 2006-10-24

**Authors:** Ting-Jung Wu, Li-Yu Lee, Chun-Nan Yeh, Pei-Yu Wu, Tzu-Chieh Chao, Tsann-Long Hwang, Yi-Yin Jan, Miin-Fu Chen

**Affiliations:** 1 Division of General Surgery, Department of Surgery, Chang Gung University College of Medicine, Chang Gung Memorial Hospital, Taoyuan, Taiwan; 2 Department of Pathology, Chang Gung University College of Medicine, Chang Gung Memorial Hospital, Taoyuan, Taiwan

## Abstract

**Background:**

Gastrointestinal stromal tumors (GISTs), the most common type of mesenchymal tumors of the gastrointestinal (GI) tract, demonstrate positive kit staining. We report our surgical experience with 100 small intestine GIST patients and identify predictors for long-term disease-free survival (DFS) and overall survival (OS) to clarify the difference between high- and low-risk patients.

**Methods:**

The clinicopathologic and follow-up records of 100 small intestine GIST patients who were treated at Chung Gung Memorial Hospital between 1983 and 2002 were retrospectively reviewed. Clinical and pathological factors were assessed for long-term DFS and OS by using a univariate log-rank test and a multivariate Cox proportional hazard model.

**Results:**

The patients included 52 men and 48 women. Their ages ranged from 27 to 82 years. Among the 85 patients who underwent curative resection, 44 (51.8%) developed disease recurrence (liver metastasis was the most common form of recurrence). The follow-up period ranged from 5 to 202 months (median: 33.2 months). The 1-, 3-, and 5-year DFS and OS rates were 85.2%, 53.8%, and 43.7%, and 91.5%, 66.6%, and 50.5%, respectively. Using multivariate analysis, it was found that high tumor cellularity, mitotic count >5/50 high-power field, and a Ki-67 index ≧10% were three independent factors that were inversely associated with DFS. However, absence of tumor perforation, mitotic count < 5/50 high power field, and tumor with low cellularity were predictors of long-term favorable OS.

**Conclusion:**

Tumors with low cellularity, low mitotic count, and low Ki-67 index, which indicate low risk, predict a more favorable DFS for small intestine GIST patients undergoing curative resection. Absence of tumor perforation with low mitotic count and low cellularity, which indicates low risk, can predict long-term OS for small intestine GIST patients who have undergone curative resection.

## Background

Mesenchymal tumors of the gastrointestinal (GI) tract are rare, comprising only 0.1% to 3% of all GI neoplasms [1]. Gastrointestinal stromal tumors (GISTs) are the most common mesenchymal tumors of the GI tract. These tumors are composed of tumor cells from the interstitial cells of Cajal [2,3], which are considered to be GI pacemaker cells.

Gain-of-function mutations in the *c-kit *proto-oncogene and overexpression of the kit protein can occur [4,5], and result in a constitutive stimulus to tumor cell growth. Because the kit tyrosine kinase inhibitor imatinib mesylate (Gleevec, formerly known as STI571, Novartis Pharma AG, Basel, Switzerland) has been shown to produce a promising clinical result in an advanced GIST patient [6], identification of GIST by kit immunopositivity has become paramount.

Surgical resection with a negative gross margin remains the mainstay of therapy for primary GISTs. However, recurrence is common, and the 5-year survival rate after complete resection ranges from 40% to 65% [7-11]. Several clinical and pathological factors that influence patient survival have been reported, but the results have varied due to variation in confirmation of kit [7-12]. Postoperative follow-up and management for small intestine GISTs after curative resection should be tailored to patients according to high- or low-risk status of the tumors. Before the era of imatinib mesylate, we identified 100 patients with primary GISTs of the small intestine using positive kit immunostaining. Our aim was to identify predictors for long-term DFS and OS for small intestine GIST patients and clarify the difference between low- and high-risk patients after curative resection.

## Methods

One hundred sixty-seven consecutive patients with mesenchymal tumors involving the small intestine were treated at the Department of Surgery, Chang Gung Memorial Hospital from January 1983 to December 2002. The study was conducted with the approval of the institutional ethics board of our hospital. We excluded (a) patients with other intra-abdominal or retroperitoneal sarcomas which directly invaded or metastasized to the small intestine (such as uterine leiomyosarcoma, gastric GISTs, retroperitoneal sarcoma, etc.), (b) patients with other concurrent malignancies at presentation, and (c) patients with incidental findings on laparotomy (most had benign leiomyoma, size < 2 cm and trace mitotic count).

Immunohistochemistry (IHC) analyses were performed to identify immunophenotypes and reviewed blindly by the authors (Wu TJ, Lee LY, Yeh CN, and Wu PU). Thirteen of 113 patients who showed negative kit immunostaining were excluded from the study including 5 patients with leiomyomas, 3 with leiomyosarcomas, 1 with a schwannoma, 1 with an inflammatory fibroid polyp, 1 with a follicular dendritic tumor, 1 with a malignant rhomboid tumor, and 1 with a malignant histocystic sarcoma. One hundred patients were diagnosed as having primary GISTs of the small intestine according to established criteria [13,14]. Clinicopathological features and follow-up status of these patients were retrospectively reviewed.

### Immunohistochemistry (IHC)

Four-μm sections from formalin-fixed, paraffin-embedded tissue were stained for ICC-associated antigens (kit and CD34) (Dako, Carpinteria, California, USA), myogenic antigen (smooth muscle actin [SMA]) (Dako, Carpinteria, California, USA), neurogenic antigen (S-100) (Dako, Carpinteria, California, USA), and tumor proliferation marker (Ki-67) (Dako, Carpinteria, California, USA). A biotin/straptavdin-peroxidase complex detection system (Dako, Carpinteria, California, USA) with diaminobenzidine (DAB) as the chromogen was used. Normal small intestine was used as the internal control for kit, SMA and S-100. The vascular endothelium was an internal positive control for CD34, and small intestine mucosa was an internal control for Ki-67. For IHC scoring, cells with positive stains below 10% were regarded as negative, 10%–50% as focally positive, and over 50% as diffusely positive. A Ki-67 index greater than 10% positively stained nuclei in 50 randomly selected high power fields (HPF) was defined as the cut line [15].

### Prognosis analysis

Patients with distant metastases or multiple small tumors involving whole small intestine (sarcomatosis) at presentation were defined as advanced cases. A curative resection was defined as the tumor having been completely removed with a negative margin macroscopically. Recurrent disease was defined as the presence of a histologically or radiographically demonstrated tumor. Distant metastatic disease was defined as disease occurring at remote structures. Regional intra-peritoneal disease was called local recurrence if it involved a solitary recurrent tumor or sarcomatosis.

The following clinical and pathological factors were chosen for survival analysis: gender, age (<60 or ≧60 years), operative time (elective, non-elective [urgent or emergent]), operative procedure, location, tumor size (<2 cm, 2–5 cm, 5–10 cm, or ≧10 cm), mesenteric involvement, local invasion to other intra-abdominal organs or peritoneum, tumor perforation, cell type (spindle or mixed with epithelioid cells), pleomorphism (low or high), mitotic count (≦5 or >5 of 50 HPF), cellularity (low or high), nuclear atypia, para-nuclear cytoplasmic vacuoles, skeinoid fibers, inflammatory cell infiltration, tumor border, tumor necrosis, mucosal invasion, ulceration, vascular proliferation, microscopic resection margins, lymphatic infiltration, immunophenotype (kit, CD34, SMA, and S-100), and Ki-67 index (<10% or ≧10%).

Data were analyzed using SPSS software (version 10.0; SPSS, Inc., Chicago, Illinois, USA). DFS and OS rates were calculated using the Kaplan-Meier method. Survival was calculated from the day of the histological diagnosis to the closing date for the evaluation of follow-up, which was December 31, 2003. The log-rank test was used for univariate analysis and Cox's proportional hazard model was used for multivariate analysis. P < 0.05 was considered significant.

## Results

### Clinical characteristics

Fifty-two males and 48 females who ranged in age from 27 to 82 years (median: 56.5 years) were symptomatic at presentation. The most common symptom was GI bleeding (n = 84), followed by abdominal pain (n = 38), anemia (n = 34), and palpable mass (n = 33). Peritonitis due to tumor perforation was found in 12 patients.

Regarding tumor location, 19 tumors were located in the duodenum (10 in the second portion; 6 in the third; and 3 in the fourth portion), 63 in the jejunum, and 17 in the ileum. One patient, who presented with multiple tumors distributed throughout the small intestine and mesentery, was classified as undetermined location. Regarding the jejunal tumors, most occurred in the proximal jejunum, with a median distance of 30 cm from the Treiz ligament. Most of the ilial tumors developed in the distal ileum, with a median distance of 60 cm from the ileocecal junction. The largest tumor diameter ranged from 3 to 30 cm (median: 8.8 cm).

Four commonly used diagnostic tools were computed tomography (CT) (78%), panendoscopy (66%), upper GI series (44%), and angiography (23%). Among the 78 patients who underwent CT, CT images revealed the presence of an intra-abdominal tumor in 71 patients, yielding a sensitivity rate of 91.0%. Routine esophagogastroduodenoscopy was performed to evaluate patients with upper GI bleeding, but only 11 of the 66 patients (16.7%) with duodenal tumors had endoscopic abnormalities. The upper GI series showed abnormal findings in 34 of the 44 tested patients (77.3%). Although half of the patients suffered from melena, only 11 of the 23 patients (43.5%) who underwent angiography had positive angiographic findings.

### Histopathologic features

Most tumors were composed of spindle cells (83%), and some were mixed with epithelioid cells (17%). No purely epithelioid picture was seen in our series. Tumor cells were mostly uniform, but some revealed significant pleomorphisms (32%). Focal nuclear atypia was a common feature (93%), with some tumors showing scattered bizarre nuclei. However, para-nuclear cytoplasmic vacuoles (10%) and skeinoid fibers (14%) were rarely found. Mitoses were common in the small intestine GISTs; 69% of patients had a mitotic count > 5 per 50 HPF and 31% of patients had a count ≦5 per 50 HPF. Coagulative necrosis (68%), mucosal ulceration (55%), and high cellularity (43%) were usually found in the larger tumors (> 5 cm). Most tumors were rich in vascular proliferation (90%), and stromal hemorrhage was a common feature of these tumors. Three patients had lymphatic infiltration.

With regard to staining distribution, most small intestine GISTs (80/100; 80%) showed diffusely positive kit immunostaining, and some showed focally positive immunostaining (20%). Most of the staining patterns showed diffuse, strong cytoplasmic positivity (69%), and some (31%) showed a mixed cytoplasmic dot-like pattern (the so-called "golgi pattern"). Sixty-eight of 100 tumors showed CD34 positivity, 35% showed myogenic differentiation that was diffusely positive in SMA, and 24% showed neurogenic differentiation that was diffusely positive for S-100 protein.

### Treatment and outcomes

Among the 100 patients, 86 underwent segmental resection of the small intestine with the tumor and the involved mesentery, peritoneum, retroperitoneum, or other contiguous intra-abdominal organs. Six patients with a tumor in the second portion of the duodenum underwent the Whipple procedure, and the other 7 patients with isolated tumors underwent local excision. The remaining patient, who presented with sarcomatosis, underwent biopsy. Elective surgery was scheduled for 72 patients; for the other 28 patients non-elective (urgent or emergent) operations were performed due to active tumor bleeding, tumor perforation-related peritonitis, or intestinal obstruction. There was no postoperative mortality in this series; hospital stays ranged from 6 to 54 days (median: 15 days). With a median follow-up of 33.2 months (range: 5–202 months), the OS rates for these 100 patients were 91.5% at 1 year, 66.6% at 3 years, and 50.5% at 5 years.

Curative resection was carried out in 85 patients. Of the 15 patients who did not undergo curative resection, 10 had liver metastasis, 4 had debulking resection due to invasion of great vessels or vital organs, and 1 had open biopsy for sarcomatosis. Patients who underwent curative resection had a significantly longer median survival of 123.3 months (mean: 97.5 months; range: 5.5–202 months) compared with 12.0 months (mean: 21.4 months; range: 4–56 months) for those who had incomplete resection or distant metastasis at presentation. Of 85 patients who underwent curative resection, 44 (51.8%) had recurrence. The median time of recurrence was 20.5 months (range: 3.7–125.1 months). The DFS rates were 85.2% at 1 year, 53.8% at 3 years, and 43.7% at 5 years.

Table 1 lists the sites of recurrence. Most cases of liver metastasis were multiple. Only 4 patients with isolated liver metastasis underwent hepatectomy. For the 44 patients with recurrence, median post-recurrent survival was 7.9 months (range: 1.2–90 months). Complete re-resection for recurrent disease was only achieved in 8 patients, with a median post-recurrence survival of 25.8 months. Chemotherapy (doxorubicin [adriamycin] and dacarbazine [DTIC]) was given to 5 patients who had a median survival of 3.6 months. Thirty-one patients with recurrence were given supportive treatment. These patients had a median survival of 5.9 months. Since 2002, imatinib mesylate (Gleevec) has been administered for unresectable GISTs in our hospital. Eight patients with unresectable recurrent disease were given this therapy.

### Survival analysis

**Figure 1:**
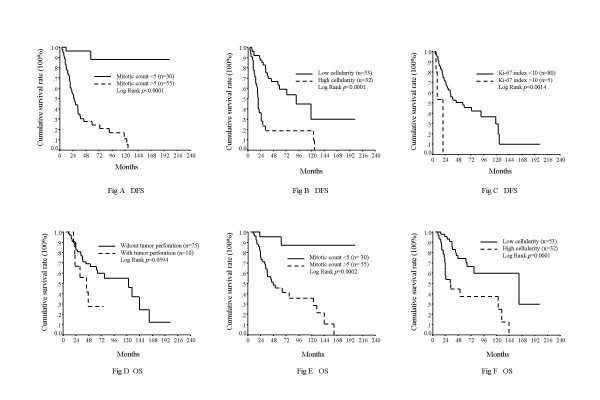
Disease-free survival for 85 patients with small bowel gastrointestinal stromal tumor (GIST) who underwent curative resection in terms of (A) mitotic count; (B) tumor cellularity; (C) Ki-67 index; and overall survival for 85 patients with small bowel GIST who underwent curative resection in terms of (D) tumor perforation; (E) mitotic count; (F) cellularity.

Regarding clinical factors, large tumor size, advanced tumor with local invasion to a contiguous organ, and presence of tumor perforation predicted poor DFS (Table 2). Among pathological factors, mitotic count > 5 per 50 HPF, high cellularity, presence of tumor necrosis, and Ki-67 index ≧10% predicted worse DFS (Tables 3 and 4). However, multivariate survival analysis revealed that tumor with high cellularity (Fig. 1A), mitotic count >5 per 50 HPF (Fig. 1B), and a Ki-67 index ≧10% (Fig. 1C) were independent factors that predicted poor DFS (Table 5).

With regard to OS, presence of tumor perforation, mitotic count >5 per 50 HPF, high cellularity, presence of tumor necrosis, vascular proliferation, lymphatic infiltration, and Ki-67 index ≧10% predicted worse OS by univariate analysis (Tables 2, 3, and 4). However, presence of tumor perforation (Fig. 1D), mitotic count >5 per 50 HPF (Fig. 1E), and tumor with high cellularity (Fig. 1F) independently predicted less favorable OS (Table 5).

## Discussions and conclusions

Gastrointestinal stromal tumors (GISTs) comprise the great majority of primary mesenchymal tumors of the gastrointestinal (GI) tract. The small intestine is the second most common site of occurrence [9-11,17-21]. Prognosis for patients with GISTs depends to some extent on the anatomic site of tumor location. According to some authors, there is a trend for small intestine tumors to have the worst prognosis and esophageal tumors the best [10,20]. However, some other authors have concluded that the behavior of GISTs is similar regardless of site [10,12,21]. Due to the controversy over the topic of anatomic variation, we focused specifically on GISTs of the small intestine. We retrospectively reviewed 100 GISTs with c-kit immunopositivity at one institution over two decades. This study is one of the largest series [20,24-26], especially before the imatinib mesylate era.

The 1-, 3-, and 5-year DFS rates following curative resection in our study were 85.2%, 53.8%, and 43.7%, respectively, which are similar to those in DeMatteo's report [11] and better than those reported by Crosby et al [20]. The OS rate was 50.5% at 5 years, which is similar to rates reported in the literature which range from 40% to 65% [8-11,22]. In this study, we used univariate and multivariate analysis to analyze DFS and OS for patients with small bowel GISTs after curative resection with regard to numerous clinicopathologic factors. The results can be used to clarify the difference between high- and low-risk patients after curative resection to further tailor follow-up programs and treatment plans.

In univariate analysis, tumor size, local invasion, tumor perforation, mitotic count, tumor cellularity, tumor necrosis, and Ki-67 index significantly influenced DFS in patients with small intestine GISTs after curative resection. Also, tumor size, tumor perforation, mitotic count, tumor cellularity, tumor necrosis, vascular proliferation, lymphatic infiltration, and Ki-67 index significantly influenced OS. Application of the multivariate Cox proportion hazard model revealed that long-term DFS was only dependent on tumor with low cellularity, low mitotic count, and low Ki-67 index. Long-term OS was dependent on no tumor perforation, low mitotic count, and low cellularity.

With regard to local invasion and tumor perforation, a tumor that has invaded a contiguous organ is considered to be advanced and associated with poor outcome [8-10]. Local invasion and tumor perforation were associated with poor DFS; although all gross disease was removed, these conditions were similar to those that occur with incomplete resections [10]. Similarly, local invasion and tumor perforation were not independent factors for DFS, but tumor perforation was an independent significant predictor for poor OS.

Tumor size was one of factors that predicted prognosis for GIST in a consensus report [13], which defined high risk if tumor size was more than 10 cm and intermediate risk if tumor size ranged from 5 to 10 cm. However, tumor size was not an independent risk factor in our study. It might be there was patient selection bias in this study, in which most of the patients who received surgical treatment were symptomatic and had a large tumor (median tumor size was 8.8 cm, range from 3 to 30 cm). It also might be biased because of the poor prognosis of small intestine GIST, in which tumor size over 5 cm is defined as probably malignant [27]. Therefore, there is no significant difference between tumors of size >10 cm and tumors from 5 to 10 cm in size.

Mitotic count < 5/50 HPF, low cellularity, no tumor necrosis, and low Ki-67 index were associated with favorable DFS, but only mitotic count >5/50 HPF and high Ki-67 index had a significant adverse influence on DFS. Some authors have also proposed that high histopathologic grade adversely affects prognosis of gastrointestinal sarcoma [8-10]. GISTs of the small intestine with histopathologic features including mitotic counts >5/50 HPF, high cellularity, absence of a predominant organoid growth pattern, absence of skeinoid fibers, presence of severe nuclear pleomorphism, presence of mucosal infilatration, and tumor cell necrosis have been significantly associated with an adverse outcome in the literature [24-26]. However, Crosby *et al*. [20] found no correlation between tumor grade and clinical behavior in 50 GISTs of the small intestine. In the present study, mitotic count >5/50 HPF was the most important independent factor predicting poor DFS and OS. Similar to Miettinen's report, kit immunopositivity, staining patterns, and histologic subtype did not correlate with prognosis [21]. Ki-67 immunoreactivity appears to be a valid measure of tumor cell proliferation and has been related to outcome in patients with GISTs in a number of studies [22,23]. Tumors with more than 10% of nuclei positive for Ki-67 analogs developed metastases more easily and had higher tumor-related mortality [15]. In the present study, Ki-67 score ≧10% was also an independent factor associated with poorer DFS, however, it was not an independent factor for OS. Mitotic count is superior to Ki-67 index analogs in the evaluation of GIST with regard to DFS and OS.

In conclusion, small tumor size with low mitotic counts and low Ki-67 index, indicating low risk, predicted more favorable DFS of small intestine GIST patients who underwent curative resection. Absence of tumor perforation with low mitotic count and low cellularity, indicating low risk, predicted long-term OS of small intestine GIST patients who underwent curative resection.

## Competing interests

The author(s) declare that they have no competing interests.

## Authors' contributions

TJW carried out manuscript writing and analyzing the data. LYL performed IHC staining and interpreting the result of IHC. CNY instructed manuscript writing. PYW helped specimen collection and IHC staining. TCC, TLH, YYJ, and MFC discussed the manuscript writing and statistical result.

## Pre-publication history

The pre-publication history for this paper can be accessed here:



## Figures and Tables

**Table 1:** Sites of first recurrence in small intestine GIST patients who underwent curative resection

Recurrence site	N	% of total recurrence
Liver	35	79.5
Any	14	31.8
Only	21	47.7
Intra-abdominal	21	47.7
Any	13	29.5
Only	8	18.2
Lung	1	2.3
Bone	1	2.3

**Table 2:** Prognostic analysis of DFS and OS for GISTs of the small intestine in terms of clinical features

Variable		Mean DFS (months; 95%CI of mean)	*P*	Mean OS (months; 95%CI of mean)	*P*
Gender	Male (N = 43)	60.0 (42.3–77.6)	0.2789	83.7 (63.0–105.5)	0.3171
	Female (N = 42)	84.3 (53.3–115.3)		108.5 (77.8–139.1)	
Age (years)	≦60 (N = 60)	66.9 (52.1–81.7)	0.3860	98.3 (79.57–117.1)	0.2646
	> 60 (N = 25)	68.8 (34.0–103.6)		93.7 (53.3–134.0)	
Operation time	Elective (N = 61)	69.4 (46.6–92.1)	0.5463	100.5 (75.8–125.2)	0.6959
	Non-elective (N = 24)	73.6 (51.3–96.0)		85.3 (61.8–108.8)	
Operative procedure	small intestine resection (N = 78)	68.3 (49.5–87.2)	0.1630	95.5 (74.8–116.2)	0.4290
	local excision (N = 7)	77.0 (54.7–99.2)		76.2 (53.1–99.3)	
Location	Duodenum (N = 17)	52.3 (31.8–72.7)	0.6391	131.4(95.6–167.1)	0.5399
	Jejunum (N = 52)	68.6 (53.2–84.0)		85.2 (67.1–103.3)	
	Ileum (N = 16)	66.8 (24.4–09.2)		96.4 (49.2–143.7)	
Tumor size	2–5 cm (N = 15)	105.1 (79.1–131.)	0.0450	135.7 (101.2–170.2)	0.2413
	5–10 cm (N = 36)	61.5 (42.5–80.6)		78.9 (53.3–104.5)	
	> 10 cm (N = 34)	67.6 (38.8–96.5)		99.8 (64.9–134.6)	
Mesenteric involvement	no (anti-mesenteric site) (N = 38)	82.5 (48.7–116.3)	0.7648	108.6 (70.2–146.9)	0.6855
	yes (mesenteric site) (N = 47)	62.3 (46.0–78.5)		89.6 (67.7–111.5)	
Local invasion	No (N = 65)	82.4 (56.5–108.3)	0.0169	102.9 (76.8–129.0)	0.7962
	Yes (N = 20)	41.9 (20.6–63.2)		90.1(60.7–119.5)	
Tumor perforation	No (N = 75)	75.7 (55.0–96.5)	0.0485	102.7 (80.8–124.8)	0.0594
	Yes (N = 10)	30.5 (15.1–45.9)		44.3 (29.7–59.0)	

DFS = disease-free survival; OS = overall survival; GISTs = gastrointestinal stromal tumors

**Table 3:** Prognostic analysis of DFS and OS for GISTs of the small intestine in terms of histopathological features

Variable		Mean DFS (months; 95%CI of mean)	*P*	Mean OS (months; 95%CI of mean)	*P*
Cell type	Spindle (N = 71)	75.1 (52.9–97.3)	0.1708	99.0(77.6–120.3)	0.3355
	Mixed with epithelioid tumor (N = 14)	59.5 (27.8–91.2)		76.8 (40.3–113.2)	
Pleomorphism	Low (N = 61)	56.6 (51.6–106.1)	0.1914	123.3 (78.1–127.5)	0.2970
	High (N = 24)	25.1 (32.2–79.6)		76.9 (52.0–101.9)	
Mitotic count	≦ 5 of 50 HPF (N = 30)	182.3 (157.0–208.6)	< 0.0001	182.4 (156.68–208.2)	0.0002
	> 5 of 50 HPF (N = 55)	43.7 (31.8–55.7)		73.8 (55.7–91.9)	
Cellularity	Low (N = 53)	103.6 (71.8–135.5)	< 0.0001	127.9 (98.5–157.4)	0.0001
	High (N = 32)	37.0 (20.1–53.8)		65.2 (42.3–88.1)	
Nuclear atypia	Absence (N = 7)	71.8 (28.8–114.7)	0.4393	123.8 (37.5–210.2)	0.4685
	Presence (N = 78)	71.4 (51.3–91.6)		95.6 (72.5–18.6)	
Para-nuclear cytoplasmic	Absence (N = 76)	69.6 (50.6–88.7)	0.4853	95.5 (74.9–116.1)	0.3995
vacuoles	Presence (N = 9)	43.7 (30.1–57.3)		54.6 (42.7–66.5)	
Skeinoid fibers	Absence (N = 72)	70.1 (50.6–89.7)	0.5636	95.3 (74.4–16.2)	0.4664
	Presence (N = 13)	50.4 (31.9–68.9)		63.8 (48.5–9.1)	
Inflammatory cell	Absence (N = 65)	72.2 (52.1–92.3)	0.5754	98.0 (76.4–119.6)	0.7927
infiltration	Presence (N = 20)	59.7 (33.6–85.9)		107.4 (54.6–103.2)	
Tumor border	Infiltrating (N = 52)	88.6 (60.9–116.2)	0.2937	106.2 (77.9–137.0)	0.1884
	well-circumscribed (N = 33)	71.7 (53.2–90.1)		130.1 (82.7–129.7)	
Tumor necrosis	Absence (N = 29)	131.1(82.3–179.9)	0.0061	148.3 (98.3–198.3)	0.0122
	Presence (N = 56)	53.8 (40.3–67.2)		82.2 (64.0–100.4)	
Mucosal invasion	Absence (N = 54)	69.7 (46.0–93.5)	0.3993	99.8 (74.6–125.1)	0.9839
	Presence (N = 31)	69.2 (47.2–91.2)		83.3 (59.9–106.78)	
Ulceration	Absence (N = 37)	78.9 (50.9–106.9)	0.4623	105.4 (76.3–134.4)	0.3830
	Presence (N = 48)	59.1 (43.6–74.6)		80.2 (62.8–97.6)	
Vascular proliferation	Absence (N = 9)	130.4 (56.8–204.0)	0.0889	82.0 (154.3–209.8)	0.0192
	Presence (N = 76)	61.9 (49.4–4.5)		83.4 (68.6 – 98.3)	
Microscopic resection	Negative (N = 81)	72.2 (52.71–91.8)	0.5059	97.9 (77.3–118.6)	0.6561
margins	Positive (N = 4)	22.8 (17.1– 28.53)		30.6 (22.0–39.1)	
Lymphatic infiltration	Absence (N = 83)	72.9 (53.3–92.6)	0.0768	99.8 (78.8–120.7)	0.0218
	Presence (N = 2)	19.8 (0.0–46.7)		29.1(14.6–43.6)	

DFS = disease-free survival; OS = overall survival; GISTs = gastrointestinal stromal tumors

**Table 4:** Prognostic analysis of DFS and OS for GISTs of the small intestine in terms of immunohistochemical features

Variable		Mean DFS (ms; 95%CI of mean)	*P*	Mean OS (ms; 95%CI of mean)	*P*
CD117 (KIT)	Focally positive (N = 12)	40.1 (18.3–61.9)	0.1276	77.8 (39.9–115.6)	0.2159
	Diffusely positive (N = 73)	74.4 (54.0–94.7)		101.6 (79.1–124.3)	
KIT immunopattern	Diffuse cytoplasmic stain (N = 55)	80.8 (54.6–107.0)	0.0981	95.5 (70.9–120.1)	0.8628
	Mixed with "golgi pattern" (N = 30)	51.6 (33.1– 70.1)		99.4 (69.1–129.7)	
CD34	Negative (N = 25)	64.8 (44.9–84.6)	0.4806	87.5 (64.7–110.4)	0.2242
	Focally positive (N = 28)	55.9 (34.6–77.2)		70.6 (48.2–93.0)	
	Diffusely positive (N = 32)	81.0 (50.6–111.4)		116.9 (84.4–149.2)	
SMA	Negative (N = 36)	76.3 (44.4–108.3)	0.1866	101.2 (70.5–132.0)	0.0637
	Focally positive (N = 17)	45.4 (24.3– 66.5)		56.8 (34.6–78.9)	
	Diffusely positive (N = 32)	82.0 (60.7–103.2)		106.8 (82.2–131.4)	
S-100	Negative (N = 34)	81.9 (49.9–113.9)	0.1274	104.9 (75.8–133.9)	0.3896
	Focally positive (N = 31)	64.5 (44.6–84.5)		79.8 (60.6–99.0)	
	Diffusely positive (N = 20)	48.4 (22.43–74.3)		78.6 (43.4–113.9)	
Ki-67 index	< 10% (N = 80)	73.6 (53.9–93.4)	0.0014	99.4 (78.5–120.2)	0.0171
	≧10% (N = 5)	13.8 (6.8–20.8)		24.0 (13.0–35.0)	

DFS = disease-free survival; OS = overall survival; GISTs = gastrointestinal stromal tumors

**Table 5:** Multivariate analysis of significant clinical, pathological, and immunohistochemical features influencing the DFS and OS in 85 patients with malignant GISTs of the small intestine who underwent curative resection

Variable	Relative risk	95% CI for relative risk	P value
**DFS**			

Tumor size (2–5, 5–10, and >10 cm)			NS
Local invasion			NS
Tumor perforation			NS
Mitotic count >5 per 50 HPF	11.36	2.653–50.0	**0.001**
High cellularity	2.004	1.036–3.876	**0.039**
Tumor necrosis			NS
Ki-67 index	3.460	1.1.195	**0.049**

**OS**			

Tumor size (2–5, 5–10, and >10 cm)			NS
Tumor perforation	3.058	1.20–7.81	**0.019**
Mitotic count >5 per 50 HPF	6.711	1.50–30.30	**0.013**
High cellularity	2.370	1.11–5.08	**0.026**
Tumor necrosis			NS
Vascular proliferation			NS
Lymphatic infilatration			NS
Ki-67 index ≧10%			NS

DFS = disease-free survival; OS = overall survival; GISTs = gastrointestinal stromal tumors; CI = confidence interval

## References

[B1] Lewis JJ, Brennan MF (1996). Soft tissue sarcoma. Curr Prob Surg.

[B2] Kindblom LG, Remotti HE, Aldenborg F, Meis-Kindblom JM (1998). Gastrointestinal pacemaker cell tumor (GIPACT): Gastrointestinal stromal tumors show phenotypic characteristics of the interstitial cells of Cajal. Am J Pathol.

[B3] Sircar K, Hewlett BR, Huizinga JD, Chorneyko K, Berezin I, Riddell RH (1999). Interstitial cells of Cajal as precursors of gastrointestinal stromal tumors. Am J Surg Pathol.

[B4] Hirota S, Isozaki K, Moriyama Y, Hashimoto K, Nishida T, Ishiguro S, Kawano K, Hanada M, Kurata A, Takeda M, Muhammad Tunio G, Matsuzawa Y, Kanakura Y, Shinomura Y, Kitamura Y (1998). Gain-of-function mutations of c-kit in human gastrointestinal stromal tumors. Science.

[B5] Lux ML, Rubin BP, Biase TL, Chen CJ, Maclure T, Demetri G, Xiao S, Singer S, Fletcher CD, Fletcher JA (2000). KIT extracellular and kinase domain mutations in gastrointestinal stromal tumors. Am J Pathol.

[B6] Joensuu H, Roberts PJ, Sarlomo-Rikala M, Andersson LC, Tervahartiala P, Tuveson D, Silberman S, Capdeville R, Dimitrijevic S, Druker B, Demetri GD (2001). Effect of tyrosine kinase inhibitor DTI571 in a patient with a metastatic gastrointestinal stromal tumor. N Engl J Med.

[B7] Akwari OE, Dozois RR, Weiland LH, Bearhrs OH (1978). Leiomyosarcoma of the small and large bowel. Cancer.

[B8] Shiu MH, Farr GH, Papchristou DN, Hajdu SI (1982). Myosarcoma of the stomach: nature history, prognostic factors and management. Cancer.

[B9] McGrath PC, Neifeld JP, Lawrence W, Kay S, Horsley JS, Parker GA (1987). Gastrointestinal sarcomas. Analysis of prognostic factors. Ann Surg.

[B10] Ng EH, Pollock RE, Munsell MF, Atkinson EN, Romsdahl MM (1992). Prognostic factors influencing survival in gastrointestinal leiomyosarcoma. Implications for surgical management and staging. Ann Surg.

[B11] DeMatteo RP, Lewis JJ, Leung D, Mudan SS, Woodruff JM, Brennan MF (2000). Two hundred gastrointestinal stromal tumors: Recurrence patterns and prognostic factors for survival. Ann Surg.

[B12] Ng EH, Pollock RE, Romsdahi MM (1992). Prognostic implications of patterns of failure for gastrointestinal leiomyosarcomas. Cancer.

[B13] Fletcher CD, Berman JJ, Corless C, Gorstein F, Lasota J, Longley BJ, Miettinen M, O'Leary TJ, Remotti H, Rubin BP, Shmookler B, Sobin LH, Weiss SW (2002). Diagnosis of gastrointestinal stromal tumors. A consensus approach. Hum Pathol.

[B14] Rudolph P, Gloeckner K, Parwaresch R, Harms D, Schmidt D (1998). Immunophenotype, proliferation, DNA-ploidy, and biological behavior of gastrointestinal stromal tumors: A multivariate clinicopathologic study. Hum Pathol.

[B15] Dougherty MJ, Compton C, Talbert M, Wood WC (1991). Sarcomas of the gastrointestinal tract: separation into favorable and unfavorable groups by mitotic count. Ann Surg.

[B16] He LJ, Wang BS, Chen CC (1988). Smooth muscle tumors of the digestive tract: report of 160 cases. Br J Surg.

[B17] Chou FF, Eng HL, Sheen-Chen SM (1996). Smooth muscle tumors of the gastrointestinal tract: analysis of prognostic factors. Surgery.

[B18] Emory TS, Sobin LH, Lukes L, Lee DH, O'Leary TJ (1999). Prognosis of gastrointestinal smooth-muscle (stromal) tumors: dependence on anatomic site. Am J Surg Pathol.

[B19] Yao KA, Talamonti MS, Langella RL, Schindler NM, Rao S, Small W, Joehl RJ (2000). Primary gastrointestinal sarcomas: analysis of prognostic factors and results of surgical management. Surgery.

[B20] Crosby JA, Catton CN, Davis A, Couture J, O'Sullivan B, Kandel R, Swallow CJ (2001). Malignant gastrointestinal stromal tumors of the small intestine: a review of 50 cases from a prospective database. Ann Surg Oncol.

[B21] Miettinen M, Kopczynski J, Makhlouf HR, Sarlomo-Rikala M, Gyorffy H, Burke A, Sobin LH, Lasota J (2003). Gastrointestinal stromal tumors, intramural leiomyomas, and leiomyosarcomas in the duodenum: a clinicopathologic, immunohistochemical, and molecular genetic study of 167 cases. Am J Surg Pathol.

[B22] Hasegawa T, Matsuno Y, Shimoda T, Hirohashi S (2002). Gastrointestinal stromal tumor: consistent CD117 immunostaining for diagnosis, and prognostic classification based on tumor size and MIB-1 grade. Hum Pathol.

[B23] Seidal T, Edvardsson H (1999). Expression of c-kit (CD117) and Ki67 provides information about the possible cell of origin and clinical course of gastrointestinal stromal tumours. Histopathology.

[B24] Brainard JA, Goldblum JR (1997). Stromal tumors of the jejunum and ileum: a clinicopathologic study of 39 cases. Am J Surg Pathol.

[B25] Tworedk JA, Appelman HD, Singleton TP, Greenson JK (1997). Stromal tumors of the jejunum and ileum. Mod Pathol.

[B26] Chang MS, Choe G, Kim WH, Kim YI (1998). Small intestinal stromal tumors: a clinicopathologic study of 31 tumors. Pathol Int.

[B27] Miettinen M, El-Rifai W, HL Sobin L, Lasota J (2002). Evaluation of malignancy and prognosis of gastrointestinal stromal tumors: a review. Hum Pathol.

